# Supplementary material for: Prior Placement of Male Urethral Slings Can Increase the Need for Revision of Artificial Urinary Sphincters
Source: J Clin Med. 2021 Dec 13;10(24):5842. doi: 10.3390/jcm10245842 (PMC8704176; doi:10.3390/jcm10245842)
Supplement: Supplementary file 1 [file jcm-10-05842-s001.zip › jcm-1486342-supplementary.pdf]

Supplementary Table S1

Supplemental Table: Multivariable Models

Erosion/Infection	Standardized Coefficients	OR	Sig.	95.0% CI	
	Beta			Lower	Upper
(Constant)		1.332	0.185	−0.04	0.206
Age >65 years	0.016	0.176	0.861	−0.12	0.143
Diabetes	0.038	0.429	0.669	−0.133	0.206
PVD	0.048	0.542	0.589	−0.257	0.451
Radiation	0.114	1.239	0.218	−0.05	0.217
Urethroplasty	−0.028	−0.314	0.754	−0.212	0.154
Prior Sling	0.04	0.445	0.657	−0.134	0.212
3.5 cm cuff	0.035	0.398	0.691	−0.121	0.183
**Revision**	Standardized Coefficients	OR	Sig.	95.0% CI	
	Beta			Lower	Upper
(Constant)		1.054	0.294	−0.059	0.193
Age >65 years	0.045	0.51	0.611	−0.1	0.169
Diabetes	0.112	1.309	0.193	−0.059	0.288
PVD	−0.069	−0.807	0.421	−0.51	0.215
Radiation	−0.037	−0.414	0.679	−0.165	0.108
Urethroplasty	0.028	0.326	0.745	−0.157	0.219
Prior Sling	0.226	2.628	0.01	0.058	0.414
3.5 cm cuff	0.138	1.614	0.109	−0.029	0.283
**Any Reoperation**	Standardized Coefficients	OR	Sig.	95.0% CI	
	Beta			Lower	Upper
(Constant)		1.867	0.064	−0.009	0.309
Age >65 years	0.048	0.54	0.59	−0.123	0.216
Diabetes	0.119	1.369	0.173	−0.067	0.37
PVD	−0.019	−0.22	0.826	−0.508	0.406
Radiation	0.056	0.631	0.529	−0.117	0.227
Urethroplasty	0.001	0.015	0.988	−0.235	0.239
Prior Sling	0.211	2.428	0.017	0.051	0.499

3.5 cm cuff	0.137	1.587	0.115	−0.039	0.354
